# Stress-Related Growth in Adolescents Returning to School After COVID-19 School Closure

**DOI:** 10.3389/fpsyg.2021.643443

**Published:** 2021-05-20

**Authors:** Lea Waters, Kelly-Ann Allen, Gökmen Arslan

**Affiliations:** ^1^Centre for Positive Psychology, Melbourne Graduate School of Education, University of Melbourne, Parkville, VIC, Australia; ^2^Faculty of Education, Monash University, Clayton, VIC, Australia; ^3^Department of Psychological Counselling and Guidance, Burdur Mehmet Akif Ersoy University, Burdur, Turkey; ^4^International Network on Personal Meaning, Toronto, ON, Canada

**Keywords:** COVID-19, strengths, positive education, posttraumatic growth, adolescents

## Abstract

The move to remote learning during COVID-19 has impacted billions of students. While research shows that school closure, and the pandemic more generally, has led to student distress, the possibility that these disruptions can also prompt growth in is a worthwhile question to investigate. The current study examined stress-related growth (SRG) in a sample of students returning to campus after a period of COVID-19 remote learning (*n* = 404, age = 13–18). The degree to which well-being skills were taught at school (i.e., positive education) before the COVID-19 outbreak and student levels of SRG upon returning to campus was tested *via* structural equation modeling. Positive reappraisal, emotional processing, and strengths use in students were examined as mediators. The model provided a good fit [*χ*^2^ = 5.37, *df* = 3, *p* = 0.146, RMSEA = 0.044 (90% CI = 0.00–0.10), SRMR = 0.012, CFI = 99, TLI = 0.99] with 56% of the variance in SRG explained. Positive education explained 15% of the variance in cognitive reappraisal, 7% in emotional processing, and 16% in student strengths use during remote learning. The results are discussed using a positive education paradigm with implications for teaching well-being skills at school to foster growth through adversity and assist in times of crisis.

## Introduction

Novel coronavirus (COVID-19) spread rapidly across the globe in 2020, infecting more than 70 million people and causing more than 1.5 million deaths at the time of submitting this paper (December 8, 2020; World Health Organization, 2020a). The restrictions and disruptions stemming from this public health crisis have compromised the mental health of young people (Hawke et al., 2020; UNICEF, 2020; Yeasmin et al., 2020; Zhou et al., 2020). A review assessing the mental health impact of COVID-19 on 6–21-year-olds (*n* = 51 articles) found levels of depression and anxiety ranging between 11.78 and 47.85% across China, the United States of America, Europe, and South America (Marques de Miranda et al., 2020). Researchers have also identified moderate levels of post-traumatic stress disorder (PTSD) in youth samples during the COVID-19 pandemic (Guo et al., 2020; Liang et al., 2020; Wang et al., 2020).

Adolescence is a critical life stage for identity formation (Allen and McKenzie, 2015; Crocetti, 2017) where teenagers strive for mastery and autonomy (Featherman et al., 2019), individuate from their parents (Levpuscek, 2006), and gravitate toward their peer groups to have their social and esteem needs met (Allen and Loeb, 2015). The pandemic has drastically curtailed the conditions for teens to meet their developmental needs (Loades et al., 2020). Gou et al. (2020, p. 2) argue that adolescents are “more vulnerable than adults to mental health problems, in particular during a lockdown, because they are in a transition phase… with increasing importance of peers, and struggling with their often brittle self-esteem.”

In addition to the researching psychological distress arising from COVID-19, it is also important to identify positive outcomes that may arise through this pandemic. Dvorsky et al. (2020) caution that research focused only on distress may create a gap in knowledge about the *resilience processes* adopted by young people. In line with this, Bruining et al. (2020, p. 1) advocate for research to keep “an open scientific mind” and include “positive hypotheses.” Waters et al. (2021) argue that researching distress during COVID-19 need not come at the expense of investigating how people can be strengthened through the pandemic. Hawke et al. (2020), for example, found that more than 40% of their teen and early adult sample reported improved social relationships, greater self-reflection, and greater self-care.

Focusing on adolescents and adopting *positive hypotheses*, the current study will examine the degree to which a positive education intervention taught at school prior to the COVID-19 outbreak had an influence on three coping approaches during remote learning (i.e., positive reappraisal, emotional processing, and strengths use) and on student levels of stress-related growth (SRG) upon returning to school.

### Can Adolescents Grow Through the COVID-19 Crisis? The Role of Positive Education

The calls for positive youth outcomes to be investigated during COVID-19 (Bruining et al., 2020; Dvorsky et al., 2020; Waters et al., 2020) align with the field of positive education. Positive education is an applied science that weaves the research from positive psychology into schools following the principles of prevention-based psychology (e.g., teaching skills that enable students to prevent distress) and promotion-based psychology (e.g., teaching skills that enable students to build well-being; Slemp et al., 2017; Waters et al., 2017).

With the WHO focusing on student well-being as a top priority during the COVID-19 crisis (World Health Organization, 2020b), positive education is an essential research area. Burke and Arslan (2020, p. 137) argue that COVID-19 could “become a springboard for positive change, especially in schools that draw on positive education research to …foster students’ social-emotional health.”

The field of positive education has developed a host of interventions that teach students the skills to support their mental health including mindfulness (Waters et al., 2015), gratitude (Froh et al., 2008), progressive relaxation (Matsumoto and Smith, 2001), sense of belonging (Allen and Kern, 2019) and, more specific to the current study, coping skills (Collins et al., 2014; Frydenberg, 2020), cognitive reframing (Sinclair, 2016), emotional management skills (Brackett et al., 2012), and strengths use (Quinlan et al., 2015). While prior research has shown that students can be successfully taught the skills to reduce ill-being and promote well-being, this research has been conducted predominantly with mainstream and at-risk students (for recent reviews, see Waters and Loton, 2019; Owens and Waters, 2020). Comparatively little research in positive education has been conducted with students who have experienced trauma (Brunzell et al., 2019) yet, in the context of a global pandemic, the risks of trauma are amplified, hence is it worth considering the role of positive education in this context.

When it comes to trauma, a number of interventions have been developed based on cognitive behavioral principles (for example, see Little et al., 2011). These interventions teach students about trauma exposure and stress responses and then show students how to utilize skill such as relaxation, cognitive reframing, and social problem-solving skills to deal with PTSD symptoms (Jaycox et al., 2010). Positive education ınterventions for trauma have been used with students who have experienced natural disasters, have been abused, have witnessed violence, or have been the victims of violent acts. These interventions have been shown to reduce depression, anxiety, and PTSD in students (Stein et al., 2003; Cohen et al., 2006; National Child Traumatic Stress Network, 2008; Walker, 2008; Jaycox et al., 2010).

The findings above, that positive education interventions help reduce the negative symptomology experienced by students in the aftermath of trauma begs the question as to whether these interventions can also promote positive changes following adversity. After first coining the term “*stress-related growth*,” Vaughn et al. (2009, p. 131) defined it as the “experience of deriving benefits from encountering stressful circumstances” and asserted that SRG goes beyond merely a state of recovery following adversity. SRG also includes the development of a higher level of ongoing adaptive functioning. Those who experience SRG come out of the adversity stronger, with a deeper sense of meaning, new coping skills, broadened perspectives and newly developed personal resources (Park and Fenster, 2004; Park, 2013).

In turning to see if positive education interventions can foster SRG, two studies were identified in the literature. Ullrich and Lutgendorf (2002) conducted a journaling intervention with undergraduate students (mean age = 20.05 years) who were asked to write about a stressful or traumatic event in their life twice a week for 1 month. Results showed that engaging in both emotion-based and cognitive-based reflection helped students see the adversity’s benefits and increase SRG. In Dolbier et al. (2010) study, college students (median age = 21 years) were placed in an intervention group or a waitlist control group. The intervention group participated in a 4-week resilience program that taught problem-focused and emotion-focused coping strategies. At the end of the program, the intervention group showed more significant increases in SRG from pre- to post-test than the waitlist control group. The findings from these two studies suggest that positive education interventions can lead to SRG. However, given that both studies used college students, there remains a gap in researcher as to whether positive interventions can promote SRG for school-aged students. As such, the question remains, “Can positive education interventions help students grow from their experience of adversity?”

Cross-sectional and longitudinal findings from traumatology, coping psychology, and adolescent psychology have shown that young people can grow through adversity. Indeed, considerable research has shown the transformative capacity within young people to use aversive experiences as a platform for growth (Levine et al., 2008; Meyerson et al., 2011). Children and teens have been found to grow following experiences such as severe illness (e.g., cancer; Currier et al., 2009), terrorist attacks (Laufer, 2006), natural disasters (e.g., floods and earthquakes; Hafstad et al., 2010), death of a parent (Wolchik et al., 2009), war (Kimhi et al., 2010), abuse (Ickovics et al., 2006), minority stress (Vaughn et al., 2009), and even everyday stressors (Mansfield and Diamond, 2017). These studies were not intervention-based but do provide consistent evidence that young people are capable of experiencing stress related growth. The findings above, showing that young people can use adversity as a springboard for growth, leads to our first hypothesis:

*Hypothesis* 1: *Adolescents will demonstrate stress-related growth during COVID-19.*

The bulk of evidence for SRG in youth samples comes from cross-sectional or longitudinal research rather than intervention-based studies. While there has been intervention-based research working with school-aged students focusing on reducing PTSD, there has been none on promoting SRG. Moreover, the CBT interventions outlined above were run with students *after the trauma* had occurred. As there is no research looking at whether learning skills through a positive education intervention *before a trauma* influences the likelihood of SRG during or following a crisis. Drawing on the principle of promotion-based positive education, the current study seeks to explore whether teaching well-being skills to students before COVID-19 was significantly related to SRG during the global pandemic. Aligning with past research findings that the coping skills existing in individuals before a traumatic event are significant predictors of growth during and after trauma (Park and Fenster, 2004; Prati and Pietrantoni, 2009; Zavala and Waters, 2020), hypothesis two is put forward:

*Hypothesis* 2: *The degree to which students were taught positive education skills at school prior to the pandemic will be directly and positively related to their SRG upon school entry.*

### Coping Approaches in Remote Lockdown and SRG Upon School Re-entry

The possibility that the stressors of COVID-19 can trigger SRG in teenagers leads to the question of what factors might increase the chances of this growth. With reduced social contact during the pandemic, Wang et al. (2020, p. 40) suggest that the development of *intrapersonal* skills are needed to optimize “psychological, emotional and behavioral adjustment.” Examples provided by Wang et al. (2020) include: (1) cognitive approaches that help students challenge unhelpful thoughts and (2) emotional approaches that give students the ability to express and handle their emotions. In research on everyday stressors with teenagers, Mansfield and Diamond (2017) found that cognitive-affective resources are significantly linked to SRG.

The coping factors examined in the current study were guided by the findings of Mansfield and Diamond (2017) together with the findings from college samples that cognitive reflection and emotional reflection (Ullrich and Lutgendorf, 2002) as well as problem-focused and emotion-focused coping (Dolbier et al., 2010) are significantly related to SRG. The recommendations of Wang et al. (2020) to investigate a student’s “psychological, emotional and behavioral adjustment” were also followed. The effect of three well-known coping skills during remote learning on SRG was examined: a cognitive skill (positive reappraisal), an emotional technique (emotional processing), and a behavioral skill – (strengths use).

Transitioning back to school, although a welcome move for many students, is still likely to be experienced as a source of distress (Capurso et al., 2020). Re-entry requires a process of adjustment and a rupture of the “new-normal” routines that students had experienced with their families at home (Pelaez and Novak, 2020). Some students may experience separation anxiety, others may be afraid of contracting the virus, and others may find the pace and noise of school unsettling (Levinson et al., 2020; Pelaez and Novak, 2020). Even for those who adjust well, a “post-lockdown school” takes time and energy to get used to – wearing masks, lining up for daily temperature checks, washing hands upon entry into classrooms, and maintaining a 1.5-meter distance from their friends are foreign for most students and will require psycho-emotional processing (Levinson et al., 2020). The better a student has coped during the period of remote learning (through positive reappraisal, emotional processing, and strengths use), the higher the chance they may have of growing through stress when they return to campus.

### Positive Reappraisal

Positive reappraisal is a meaning-based, adaptive cognitive process that motivates an individual to consider whether a good outcome can emerge from a stressful experience (Carver et al., 1989; Folkman and Moskowitz, 2000). Positively reappraising a stressful experience in ways that look for any beneficial outcomes (Garland et al., 2011) has been shown to make people more aware of their values in life and to act upon those values (Folkman and Moskowitz, 2000), thus, in doing so, it is linked to a deeper sense of meaning in life emerging from the stressor (Rood et al., 2012a). Positive reappraisal has been shown to reduce distress and improve mental health outcomes across various crises such as chronic illness, war, and rape (Sears et al., 2003; Helgeson et al., 2006). Concerning the COVID-19 crisis, Xie et al. (2020) assert that an optimistic outlook may be critical. The reverse pattern has also been found in two student samples (Liang et al., 2020; Ye et al., 2020) during the coronavirus crisis. Negative rumination (i.e., repeated negative thoughts about the virus) has been related to higher distress levels. Learning how to re-construct obstacles into opportunities during COVID-19 (e.g., “I miss seeing my teachers in person, but I am learning to be a more independent student”) can help young people to emerge from the crisis with new mindsets and skillsets. This logic leads to the third hypothesis of the current study:

*Hypothesis* 3: *Higher use of positive reappraisal during remote learning will be significantly related to higher levels of stress-related growth when students return to school.*

### Emotional Processing

Emotional processing is described as the technique of actively processing and expressing one’s emotions during times of stress (in contrast to avoidance; Stanton et al., 2000). Emotional processing is a positive factor in helping children cope with and grow through adverse events such as grief (McFerran et al., 2010), identity conflict (Davis et al., 2015), and natural disasters (Prinstein et al., 1996). To date, the role of emotional processing during a pandemic has not been explicitly studied; however, there is indirect research to suggest the value of this coping approach. For example, students in Chen et al. (2020) study who knew how to manage their stress levels displayed fewer symptoms of depression during COVID-19. Similarly, in Duan et al. (2020) study, emotion-focused coping during the coronavirus crisis was significantly related to anxiety levels in students from Grade 3 to Grade 12. These findings lead to Hypothesis four:

*Hypothesis* 4: *Higher use of emotional processing techniques during remote learning will be significantly related to higher levels of stress-related growth when students return to school.*

### Strengths Use

The third coping factor to be examined in the current study is the skill of strengths use. Strengths are defined as positive capacities and characteristics that are energizing and authentic (Peterson and Seligman, 2004). Strengths use is described by Govindji and Linley (2007) as the extent to which individuals put their strengths into actions and draw upon their strengths in various settings. Shoshani and Slone (2016) showed that strengths have a moderating role in the relationship between political violence and PTSD for young people exposed to lengthy periods of war and political conflict. In an adult sample, strengths were found to enhance PTG in earthquake survivors (Duan and Guo, 2015). In the current COVID-19 pandemic, Rashid and McGrath (2020, p. 116) suggest that “using our strengths can enhance our immunity to stressors by building protective and pragmatic habits and actions.” Adding to this, research shows that strengths use leads to an increased sense of control/self-efficacy in young people (Loton and Waters, 2018), which may be an important outcome to combat the “uncertainty distress” (Freeston et al., 2020) that many young people are currently feeling (Demkowicz et al., 2020). The research and reasoning outlined above about strengths use has been used to formulate Hypothesis five.

*Hypothesis* 5: *Higher strengths use during remote learning will be significantly related to higher levels of stress-related growth when students return to school.*

Having established that the three coping approaches above are likely to foster SRG during COVID-19, the final question remaining is whether positive education interventions can increase the use of these coping approaches. Garland et al. (2011) and Pogrebtsova et al. (2017) found that mindfulness interventions increase positive reappraisal. In a related outcome, positive education interventions have been shown to help students better understand their explanatory styles (i.e., how they interpret adversity; Quayle et al., 2001; Rooney et al., 2013). Adding to this, emotional processing is significantly enhanced in students due to undertaking various positive education interventions (Qualter et al., 2007; Brackett et al., 2012; Castillo et al., 2013). Finally, positive education interventions have been shown to increase strengths use (Marques et al., 2011; Quinlan et al., 2015; White and Waters, 2015). These findings inform our final two study hypotheses.

*Hypothesis* 6: *The degree to which students were taught positive education skills at school prior to the pandemic will be directly and positively related to their use of positive reappraisal, emotional processing, and strengths use during remote learning.*

*Hypothesis* 7: *The degree to which students were taught positive education skills at school prior to the pandemic will be indirectly and positively related to SRG upon school entry via their use of positive reappraisal, emotional processing, and strengths use during remote learning.*

## Materials and Methods

### Participants

After receiving Ethics approval from the Human Ethics Research Committee at Monash University, data were collected from 404 students at a large independent school in New South Wales, Australia. Participants were recruited from Grades 7 to 12 and ranged in age from 11 to 18 (*M* = 14.75, *SD* = 1.59; 50.2% female/46.8% male and 3% identified as non-/other gendered or declined to answer). The vast majority of the sample (93.1%) listed English as their primary language. Prior to conducting the survey, parents were sent information packages explaining the nature of the research, resources available to students feeling distress, security and anonymity of the data collected, and the opt-out process. Participation was voluntary and students could opt out at any time.

Students in the current study were part of a whole-school positive education intervention that focuses on six key pathways to well-being: strengths, emotional management, attention and awareness, relationships, coping, and habits and goals (Waters, 2019; Waters and Loton, 2019). The first letter of each of these six pathways forms the acronym “SEARCH.” In 2019, all teachers at the school were trained in the “SEARCH” pathways and given activities to run in classrooms that help students learn skill that allow them to build up the six pathways of strengths (e.g., strengths pathways: strength surveys and strengths challenges), managing their emotions (e.g., learning how to label the full spectrum of emotions and identifying emotions through a mood-meter), focusing their attention (e.g., mindfulness), building their relationships (e.g., active-constructive responding), coping (e.g., cognitive reframing and breathing techniques), and building habits and setting goals (e.g., if-then intentions).

### Measures

#### Positive Education

Students rated the degree to which they had been taught positive education skills at school prior to the COVID-19 pandemic along the six pathways of the SEARCH framework. Students rated the degree to which they had been taught how to use their strengths, manage their emotions, and build their capacity to have awareness and so on, prior to COVID-19. There was one item per SEARCH pathway (e.g., “Prior to COVID-19, to what degree did your school teach you about how to understand and manage your emotions?”). The alpha reliability for this scale was 0.91.

#### Emotional Processing

Students rated the degree to which they engaged in emotional processing techniques (“I took time to figure out what I was feeling,” “I thought about my feelings to get a thorough understanding of them,” etc.) during the COVID-19 pandemic and lockdown using the 4-item scale Emotional Processing Scale (Stanton et al., 2000). Answers were given on a 4-point scale from “I didn’t do this at all” to “I did this a lot.” The internal reliability of the scale was *α* = 0.78.

#### Positive Reappraisal

Positive reappraisal was measured using the 4-item “Positive Reinterpretation and Growth Scale” of the COPE inventory (Carver et al., 1989). Students were asked to rate the degree to which they engaged in positive reappraisal techniques (“I looked for something good in what was happening,” “I learned something from the experience,” etc.) during the COVID-19 pandemic and lockdown. Answers were given on a 4-point scale from “I didn’t do this at all” to “I did this a lot.” The internal reliability of the scale was *α* = 0.82.

#### Strengths Use

Students rated the degree to which they used their strengths during the remote learning using an adapted three-item version of the Strengths Use Scale (Govindji and Linley, 2007), a 14-item self-report scale designed to measure individual strengths use (e.g., items included “During remote learning and family lockdown I had lots of different ways to use my strengths,” “During remote learning and family lockdown I achieved what I wanted by using my strengths,” etc.). Answers were given on a 5-point scale from 1“Not at all” to 5 “A lot.” The internal reliability of the scale was *α* = 0.89.

#### Stress-Related Growth

Using an abbreviated Stress-Related Growth Scale (Vaughn et al., 2009), students were asked to think about whether their experience with COVID-19 changed them in any specific ways, including internal growth (“I have learned to deal better with uncertainty,” “I learned not to let small hassles bother me the way they used to,” etc.) and social growth (“I reached out and helped others,” “I have learned to appreciate the strength of others who have had a difficult life,” etc.). Answers were given on a 5-point Likert scale from “Not at all” to “A lot.” The internal reliability of the scale was *α* = 0.85.

### Procedure

For students who elected to participate in this study, the school distributed the survey *via* an email link on the Qualtrics platform distributed by the teachers during the students’ mentor time. The first screen of the form provided information on the survey and reminded students that they could opt out or stop at any time. If distressed, several resources were made available, including teachers at the school, parents, and helplines. Teachers were present during the entire duration of the survey to provide clarification on instructions and/or support for students feeling distressed.

The data collected from the survey were anonymized and shared with the participating school administrators, and this was clearly stated to all participants of the study, including teachers, parents, and students. No personally identifiable information was made available in the data asset provided to the school. The original data source from the survey will be stored in a secure, password-protected file at Monash University for 5 years.

Through the survey, students were asked to reflect upon three different points in time: before school closures, during school closures, and after return to school. More specifically, students were asked to reflect on the positive education taught by their school prior to COVID-19. They were asked to reflect on what actions they took during COVID-19 lockdown to thrive cognitively (positive reappraisal), emotionally (emotional processing), and behaviorally (strengths use). And upon return to school, students were asked to reflect upon what they had learned as a result of the COVID-19 pandemic and lockdown (SRG).

#### Data Analysis

We carried out a two-step analytic approach to examine the association between positive education indicators and student levels of SRG upon returning back to campus during the COVID-19 outbreak. Observed scale characteristics were first performed to investigate descriptive statistics and the assumptions of analysis. Normality assumption was checked using kurtosis and skewness scores, with their cut points for the normality. Skewness <|2| and kurtosis scores <|7| suggest that the assumption of normality is met (Curran et al., 1996; Kline, 2015). Pearson correlation was additionally used to examine the association between the variables of the study.

Following this, structural equation modeling was used to test the mediating effect of positive reappraisal, emotional processing, and strengths use during the period of remote learning in the association between positive education (i.e., the degree to which well-being skills were taught at school prior to the COVID-19 outbreak) and SRG upon returning back to campus. Common data-model fit statistics and squared multiple correlations (*R*^2^) were examined to evaluate the results of structural equation modeling. Tucker-Lewis index (TLI) and comparative fit index (CFI) scores between 0.90 and 0.95 indicate adequate model fit, whereas their scores ≥0.95 provide a good or close data-model fit. The root mean square error of approximation scores (RMSEA; with 90% confidence interval) and the standardized root mean square residual (SRMR) between 0.05 and 0.08 are accepted as an adequate model fit, while those scores ≤0.05 indicate a close model fit (Hu and Bentler, 1999; Hooper et al., 2008; Kline, 2015). The results were also interrelated using the squared multiple correlations (*R*^2^) with: <0.13 = small, 0.13–0.26 = moderate, and ≥0.26 = large (Cohen, 1988). All data analyses were performed using AMOS version 24 and SPSS version 25.

## Results

### Observed Scale Characteristics and Inter-correlations

A check of observed scale characteristics showed that all measures in the study were relatively normally distributed, and that kurtosis and skewness scores ranged between −0.80 and 0.47 (see Table 1). As shown in Table 1, correlation analysis found that teaching positive education prior to COVID-19 had positive correlations with the way students coped during remote learning (positive reappraisal, emotional processing, and strengths use) and with SRG when returning to campus. Additionally, positive reappraisal, emotional processing, and strengths use were moderately to largely, positively associated with SRG.

**Table 1:** Descriptive statistics and correlation results.

Scales	Range	*M*	*SD*	Skew.	Kurt.	α	1	2	3	4	5
1 Positive education	7–35	20.21	7.68	0.27	−0.71	0.91	–	0.26^**^	0.39^**^	0.40^**^	0.40^**^
2 Emotional processing	4–16	8.78	3.10	0.38	−0.36	0.78		–	0.57^**^	0.48^**^	0.48^**^
3 Positive reappraisal	4–16	10.27	2.85	0.01	−0.56	0.82			–	0.59^**^	0.59^**^
4 Strengths use	4–15	8.28	3.14	0.27	−0.52	0.89				–	0.51^**^
5 Stress-related growth	6–30	16.65	5.65	0.51	−0.01	0.85					–

** Correlation is significant at the 0.001 level (two-tailed).

### Mediation Analyses

Several structural equation models were employed to analyze the mediating effect of positive reappraisal, emotional processing, and strengths use in the relationship between positive education and SRG. The first model, which was conducted to test the mediating role of emotional processing indicated good data-model fit statistics (*χ*^2^ = 1.20, *df* = 1, *p =* 0.273, RMSEA = 0.069 [90% CI for RMSEA: 0.00–0.13], SRMR = 0.010, CFI = 99, and TLI = 0.99). Standardized regression estimates revealed that positive education was a significant predictor of emotional processing and SRG. Moreover, emotional processing significantly predicted youths’ SRG. The indirect effect of positive education on SRG through emotional processing was significant, as seen in Table 2. Positive education accounted for 7% of the variance in emotional processing, and positive education and emotional processing together explained 41% of the variance in SRG. These findings demonstrate the partial mediating effect of emotional processing on the link between positive education and student SRG upon returning to campus during the COVID-19 outbreak.

**Table 2:** Standardized indirect effects.

Path	Effect	*SE*	BootLLCI	BootULCI
**Emotional processing**				
Positive education⟶ Stress-related growth	0.12	0.03	0.08	0.18
**Positive reappraisal**				
Positive education⟶ Stress-related growth	0.22	0.03	0.17	0.29
**Strengths use**				
Positive education⟶ Stress-related growth	0.20	0.03	0.14	0.26
**All mediators**				
Positive education⟶ Stress-related growth	0.28	0.04	0.21	0.35

Number of bootstrap samples for percentile bootstrap confidence intervals: 10,000 with 95% bias-corrected confidence interval.

The second model, which was conducted to test the mediating effect of positive reappraisal, indicated excellent data-model fit statistics (*χ*^2^ = 0.76, *df* = 1, *p* = 0.382, RMSEA = 0.00 [90% CI for RMSEA: 0.000–0.12], SRMR = 0.010, CFI = 1.00, and TLI = 1.00). Positive education had a significant predictive effect on positive reappraisal and SRG. Positive reappraisal also significantly predicted youths’ SRG. The indirect effect of positive education on SRG through positive reappraisal was significant, as seen in Table 2. Positive education accounted for 15% of the variance in positive reappraisal, and positive education and positive reappraisal together explained 50% of the variance in SRG. Consequently, the findings of this model indicated the partial mediating effect of positive reappraisal in the relationship between positive education and student SRG.

The third model, which was carried out to examine the mediating effect of strengths use, indicated excellent data-model fit statistics (*χ*^2^ = 0.24, *df* = 1, *p* = 0.626, RMSEA = 0.00 [90% CI for RMSEA: 0.000–0.10], SRMR = 0.010, CFI = 1.00, and TLI = 1.00). Positive education significantly predicted strengths use and SRG. Strengths use also had a significant predictive effect on student SRG. The indirect effect of positive education on SRG through strengths use was significant, as seen in Table 2. Positive education accounted for 16% of the variance in positive reappraisal, and positive education and strengths use together explained 40% of the variance in the SRG of youths. These results indicate the partial mediating effect of strengths on the association between positive education and student SRG.

**Figure 1:**
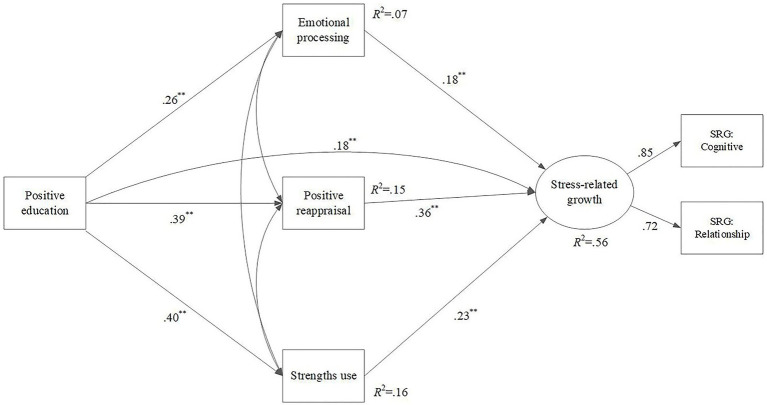
Structural equation model indicating the relationship between the variables of the study. ^**^p < 0.001.

The final and main model tested the mediating effects of emotional processing, positive reappraisal (see Figure 1), and strengths use yielded data-model fit statistics (*χ*^2^ = 5.37, *df* = 3, *p* = 0.146, RMSEA = 0.044 [90% CI = 0.00–0.10], SRMR = 0.012, CFI = 99, and TLI = 0.99). Standardized estimates showed that positive education had significant and positive predictive effect on emotional processing, positive reappraisal, strengths use, and SRG. Furthermore, SRG was significantly predicted by emotional processing, positive reappraisal, and strengths use. In this model, all variables together explained 56% of the variance in the SRG (before including mediators = 21%). The indirect effect of positive education on SRG through the mediators was significant, as shown in Table 2. Taken together, the findings of this model demonstrate the partial mediating effect of emotional processing, positive reappraisal, and strengths use in the relationship between positive education and the SRG of students.

## Discussion

As the COVID-19 global health disaster continues to unfold across the world, researchers are rushing to quantify adolescent psychological distress (Marques de Miranda et al., 2020). Such investigation is important because children and teenagers have been identified as a particularly vulnerable group within our society during the pandemic (Guo et al., 2020). Research into teen distress is crucial but it need not come at the expense of learning about the positive outcomes that young people might experience in a pandemic. In this time of global crisis triggered by the COVID-19 pandemic, studies investigating how young people can come out stronger is vitally important given the findings from earlier pandemics that psychopathology and PTSD can last for up to 3 years post the pandemic (Sprang and Silman, 2013).

The current study adopted a positive education approach, specifically a promotion-based orientation, to explore a range of factors that increase a student’s likelihood of experiencing SRG. Previous research has shown that young people can, and do, grow through adverse experiences (Salter and Stallard, 2004). This same finding was shown in the current study where the mean score for cognitive/affective growth (e.g., “I learned not to let small hassles bother me the way they used to”) was 2.57 + 1.2 out of 5 and the mean score for social growth (e.g., “I have learned to appreciate the strength of others who have had a difficult life”) was 2.96 + 1.3. These scores are higher than other youth samples who have experienced minority stress (cognitive/affective growth 2.30 + 0.65/social growth 2.45 + 0.63; Vaughn et al., 2009) and those who reported on everyday stressors (cognitive/affective growth 2.10 + 0.60/social growth 2.15 + 0.63; Mansfield and Diamond, 2017).

In addition to demonstrating that teenagers can experience growth during the global pandemic, this study also sought to explore the degree to which a set of cognitive, emotional, and behavioral coping skills used during remote learning could predict SRG upon school re-entry. All three coping skills significantly predicted the degree to which students reported they had grown through COVID-19. Positive reappraisal had the strongest effect, followed by strengths use and then emotional processing.

Positive reappraisal is a meaning-based, cognitive strategy that allows an individual to “attach a positive meaning to the event in terms of personal growth” (Garnefski and Kraaij, 2014, p. 1154). This cognitive coping skill has been shown to be an adaptive way to help teenagers deal with a range of distressing situations, including being bullied (Garnefski and Kraaij, 2014) and coping with loss, health threats, and relational stress (Garnefski et al., 2003). Interestingly, Garnefski et al. (2002) found that adolescents did not use positive reappraisal as frequently as adults, suggesting that this is a skill that is worth teaching in the positive education curriculum in the future. To this end, Rood et al. (2012b, p. 83) explored whether positive reappraisal could be experimentally induced in teenagers and found that it could by asking them to think of stressful event in their lives and then “try to think about the positive side effects of the stressful event. Examine what you have learned, and how it has made you stronger.” Moreover, Rood et al. (2012b) found that of the four cognitive coping skills induced in teenagers (rumination, distancing, positive reappraisal, and acceptance), it was positive reappraisal that had the largest impact on well-being.

The degree to which students reported using their strengths during remote learning was also significantly, positively related to the amount of SRG they reported once back on campus. Strengths use has been positively associated with a host of well-being indicators for adolescents during mainstream times (i.e., non-crisis times) including life satisfaction (Proctor et al., 2011), subjective well-being (Jach et al., 2018), and hope (Marques et al., 2011). In the context of adversity, strengths have also been shown to assist coping. For example, Shoshani and Slone (2016) found that strengths were inversely related to psychiatric symptoms and PTSD in teenagers experiencing war and political violence. In times of natural disaster, Southwick et al. (2016) argued that strengths help young people deal with the crisis adaptively and find solutions for the obstacles. In the COVID-19 pandemic, Bono et al. (2020) found that the strengths of grit and gratitude fostered resilience and impacted grades in university students. Our study found that strengths use was a significant predictor of the degree to which teenagers experienced SRG, suggesting that teaching students to identify and use their strengths will be beneficial in preparing them to grow through the current pandemic and in future times of adversity.

The third coping skill tested in this study was that of emotional processing, which is characterized by the conscious way a person acknowledges and handles intense emotions that come along with a distressing event or experience (Stanton and Cameron, 2000). Park et al. (1996) found that seeking emotional support from others and venting one’s emotions were significantly related to SRG in university students. The intensity, uncertainty, and fear surrounding the global pandemic has triggered youth depression and anxiety (Ellis et al., 2020) as well as heightened the experience of many, sub-clinical, negative emotions such as loneliness, frustration, anger, and hopelessness (Garbe et al., 2020; Horita et al., 2021). With this in mind, it is easy to see why it is important for students to have the skills to adaptively work through their emotions each day. The degree to which students identified, validated, and expressed, their emotions was a significant predictor of SRG in the current study.

The positive education intervention tested in this study was a factor that significantly predicted the use of the three coping skills during remote learning. More specifically, the more that students reported they had been taught the skills for increasing their levels of “SEARCH” (strengths, emotional management, attention and awareness, relationships, coping, and habits and goals; Waters, 2019; Waters and Loton, 2019), the more they were likely to utilize adaptive coping skills during remote learning. The positive education intervention in this study predicted 15% of the variance in positive reappraisal, 7% of the variance in emotional processing, and 16% of the variance in strengths use. Additionally, the positive education intervention had an indirect effect on SRG through its impact on the three coping skills. The degree to which students felt they had learnt well-being skills prior to COVID-19 explained 21% of SRG (before including mediators) and 56% of the variance when including the three coping skills. In non-pandemic periods, positive education interventions have been shown to promote coping skills and well-being in students. The current study shows the value of positive education interventions during times of crisis suggesting that this field plays an important role in preparedness and prevention for future challenges (Almazan et al., 2018, 2019; Mohammadinia et al., 2019).

The role of positive education interventions for a student’s ability to grow from the adversity created by COVID-19 has important implications for schools moving forward in the pandemic. At the time of writing this paper, the United States of America, the United Kingdom, Malaysia, and parts of Europe have all announced a second wave of lockdown (Khan et al., 2020; Kupferschmidt, 2020; Once more with dread, 2020) and many schools are moving back into remote learning, with no certainty as to when students will be back on campus. Schools, through a positive education approach, can help students to enhance their *psychological immunity* and *psychological flexibility* to this virus in order to cope with the multiple stages of the pandemic they are cycling through (Arslan et al., 2020; Arslan and Allen, 2021). Positive education interventions equip students with the vital skills that enable them to build healthy relationships with others – crucial for building social support networks, strong student-teacher alliances (Allen et al., in press), and fostering a sense of belonging to their school (Frydenberg et al., 2012; Allen et al., 2017, 2018, 2019). Given the challenges of social connection occurring during the pandemic, and especially during remote learning, these skills are vital to retain that sense of social connection needed by teens.

Capurso et al. (2020, p. 66) assert that it is important for schools “to instigate a sense-making process in children by providing an arena where they can process critical events connected to the COVID-19 pandemic at both an emotional and a cognitive level, thereby building up their resilience and minimize the risk of long-lasting trauma.” Several other authors have also called for the need for school-family/school-community partnerships to build “resilient systems” around young people during times of crisis (Prinstein et al., 1996; Brock and Jimerson, 2004; Masten and Motti-Stefanidi, 2020). Dvorsky et al. (2020, p. 1) call for “school-wide wellness supports” that promote “adaptive coping and resilience-promoting processes.” Findings from the current study suggest that teaching students how to engage in positive reappraisal, emotional processing, and strengths use will increase their chances of growing through the stress they are experiencing.

The coping skills tested in this study could be seen to provide a “psychological formula” to help students validate the negatives (e.g., through emotional processing) and identify the positives (e.g., through positive reappraisal). Striking the balance between owning the pain and seeing the positives was a key factor in adversarial growth experienced by SARS patients (Cheng et al., 2006). Adding strengths use to positive reappraisal and emotional processing provides the action element to round out a “cognitive-emotion-behavioral approach” recommended by Wang et al. (2020) and helps students to use their strengths to regain a sense of agency in a time of uncertainty. According to Rashid and McGrath (2020, p. 119), focusing on strengths makes the negative “less powerful” and “reminds us that we have our personal strengths that can carry us through the crisis.”

### Limitations

The results of this study must be considered within its limitations. First, the sample was obtained from only one school which was an independent grammar school in Australia (predominantly English speaking). This may limit the generalizability of the findings for students who attend schools from other sectors such as public/Government schools or other faith-based schools and for students in non-WEIRD^1^ contexts. Second, the retrospective design means there is potential that the results were influenced by recall bias. Students were asked to complete a single survey recalling two previous time points (prior to the onset of the pandemic, during remote learning) and their SRG in the current return to campus. It is possible that some students either underrated or overrated the degree to which they learned positive education. Under ideal circumstances, student ratings of the skills they were learning through the positive education would have been collected *prior* to the pandemic; however, because the COVID-19 pandemic was not anticipated, the current design relied on retrospective recall.

Another consideration is the long-term duration of COVID-19 itself. As the COVID-19 pandemic continues through the time of writing this paper (December 2020), and is expected to go on for some time longer, the lasting effects of the pandemic may impact the nature of SRG experienced in students. The role that the duration of this crises plays on SRG is currently unknown, which means that there is benefit in exploring whether SRG grows, stalls, or reverses as the pandemic continues.

## Conclusion

To assist adolescents during COVID-19, researchers need to provide a comprehensive and well-rounded understanding of their experiences. The addition of positive education science to the current COVID-19 research helps to provide researchers, teachers, and school psychologists with a deeper understanding of the factors that promote internal resources, strengths, and positive outcomes for teens.

## Data Availability Statement

The raw data supporting the conclusions of this article will be made available by the authors, without undue reservation.

## Ethics Statement

The studies involving human participants were reviewed and approved by Monash University Human Research Ethics Committee. Implied consent was used and approved meaning that written informed consent from the participants’ legal guardian/next of kin was not required to participate in this study in accordance with the national legislation and the institutional requirements.

## Author Contributions

LW led the research, conceptualized the study, led the study design, sourced the bulk of the research measures, recruited the research site school, and wrote the abstract, introduction, discussion, and references. K-AA led the Ethics approval process, sourced some of the research measures, and wrote the materials and methods and limitations. GA sourced some of the research measures, led the statistical analysis, and wrote the results section. All authors contributed to the article and approved the submitted version.

### Conflict of Interest

The authors declare that the research was conducted in the absence of any commercial or financial relationships that could be construed as a potential conflict of interest.

## Footnotes

^1^WEIRD: Western, educated, industrialized, rich and democratic.
